# Impact of Physical Exercise on Adult Asthma Control: A Randomized Clinical Trial

**DOI:** 10.3390/healthcare13202634

**Published:** 2025-10-20

**Authors:** Sarah Micozzi, Pilar Gajate Fernández, Paula Sánchez López, Jimena Laiseca García, Francisco Javier Pérez Rivas

**Affiliations:** 1 Allergy Department, University Hospital Rey Juan Carlos, 28933 Móstoles, Spain; pilar.gajate@hospitalreyjuancarlos.es; 2 Health Research Institute-Fundación Jiménez Díaz University Hospital, Universidad Autónoma de Madrid, 28040 Madrid, Spain; 3 Allergy Department, University Hospital of Tajo, 28300 Aranjuez, Spain; 4 Allergy Department, University Hospital of Fuenlabrada, 28942 Fuenlabrada, Spain; jimenalaiseca91@gmail.com; 5 Department of Nursing, Faculty of Nursing Physiotherapy and Podiatry, Complutense University of Madrid, 28040 Madrid, Spain; frjperez@ucm.es; 6 Health Research Institute 12 de Octubre Hospital (Imas12), 28041 Madrid, Spain

**Keywords:** sedentary behavior, asthma, asthma control, physical activity, mild asthma, health education, randomized controlled trial

## Abstract

**Background:** Most research on asthma and physical exercise is complex, costly, and often inconclusive, leading to minimal mention of exercise in international asthma management guidelines. Patients with mild asthma are frequently excluded from clinical trials, which focus on more severe cases, resulting in a lack of scientific evidence for this population. **Objective:** This study aimed to evaluate the effectiveness of a 3-month health intervention program promoting unstructured physical activity to improve asthma control, defined as a decrease of 0.5 points in the Asthma Control Questionnaire (ACQ-5). **Methods:** the study was designed as an open-label, two-arm randomized clinical trial involving sedentary asthmatic patients with intermittent to moderate persistent asthma. Participants were divided into an intervention group that attended three workshops based on World Health Organization recommendations for physical activity and a control group that continued their usual activities. All participants underwent three medical visits to assess variables such as asthma control, quality of life, rescue medication use, exacerbations, average steps taken, and respiratory function. **Results:** A total of 52 patients were recruited and randomized (26 patients per group), (73.1% women), with 48 completing the study (24 patients per group). The intervention group showed significant improvements in ACQ-5 scores (*p* = 0.035), mini-AQLQ score (*p* = 0.017), and average daily steps (*p* < 0.001). Significant differences were also observed between groups regarding respiratory function (*p* = 0.04) and average daily steps (*p* = 0.01). **Conclusions:** in sedentary asthmatic patients, including those with milder profiles, implementing low-resource physical exercise interventions significantly improved the average steps taken and respiratory function, while asthma control and rescue medication use showed a positive trend.

## 1. Introduction

Research on asthma and physical exercise has evolved in various ways, ranging from strict multidisciplinary respiratory rehabilitation programs [1] to more flexible interventions with different types of exercise like yoga [2]. This diversity has resulted in a collection of heterogeneous clinical trials that are challenging to compare, often leading to inconclusive results [3]. Many patients receive only a clinical diagnosis of asthma without reliable tests for definitive diagnosis or severity staging [4]. When staging is conducted, trials frequently exclude patients with milder forms of asthma [5], leaving a gap in data for this population.

While structured multidisciplinary rehabilitation programs exist, they face significant limitations, including high dropout rates and low adherence [6]. These programs are resource-intensive and costly, making them applicable to only a limited number of patients [7]. In contrast, simpler programs that promote standardized physical activity tend to have better adherence but struggle to accurately measure physical activity levels and sedentary behavior [3].

The literature presents heterogeneous data regarding the types of physical exercise beneficial for asthma. However, aerobic exercise, particularly when combined with respiratory training, appears to be the most effective in improving respiratory function [5,8]. Key findings indicate that less active asthmatic patients experience more exacerbations and have more unscheduled medical visits compared to their more active counterparts, who generally exhibit better clinical profiles [3]. Additionally, research has explored whether physical exercise can prevent the onset of asthma [9].

The pillars of asthma prevention and management include dietary choices, health education, weight control, and psychological support programs [10]. These strategies have varying impacts at different life stages, but physical exercise seems to have the most lasting effect on asthma prevention and management over a lifetime [10].

Recognizing the crucial role of physical activity in asthma control and the challenges posed by existing interventions, a clinical trial is proposed to evaluate the effectiveness of a program aimed at promoting physical exercise among a well-characterized sedentary asthmatic population, including those with less advanced stages of the disease. The study’s hypothesis is that in sedentary asthmatic patients, the implementation of daily physical exercise consisting of more than 7,000 steps per day, and established through an educational program, improves asthma control compared to asthmatic patients who remain sedentary. The primary objective of the study is to assess the efficacy of a 3-month health intervention program on unstructured physical activity, aiming for an improvement in asthma control defined as a decrease of 0.5 points [11] in the Asthma Control Questionnaire (ACQ-5) [12,13].

Secondary objectives will evaluate the intervention’s effectiveness on lung capacity, quality of life, use of rescue medication, and the frequency of exacerbations. Finally, the study will assess how sociodemographic characteristics and the clinical and functional status of patients influence the intervention’s effectiveness.

## 2. Materials and Methods

### 2.1. Trial Design

A randomized, open-label, two-arm, longitudinal, and prospective clinical trial. The clinical trial has been registered at https://clinicaltrials.gov/ with the number NCT05900120, on 1 June 2023, prior to the start of patient recruitment. The trial was reported in accordance with the CONSORT 2010 guidelines; the completed checklist is available in the Appendix A Table A1.

### 2.2. Setting and Population

The study was conducted in the Allergy Clinic of the Rey Juan Carlos University Hospital in Móstoles, with patients aged between 18 and 64 years, who attended the clinic consecutively from 5 June 2023 until the required sample size was reached on 24 November 2023. All patients had to meet the established inclusion and exclusion criteria. The follow-up finished on 28 February 2024.

#### 2.2.1. Inclusion Criteria

- Patients with a diagnosis of asthma according to the criteria of the Spanish Asthma Management Guide [14];
- Severity: Intermittent asthma, mild persistent asthma, and moderate persistent asthma [14];
- ACQ-5 ≤ 1.5 at the time of recruitment (well or partially controlled asthma);
- International Physical Activity Questionnaire Short Form (IPAQ-SF) [15] category 1 (low) and verified performance of <7000 steps daily (patients below the WHO physical activity recommendations for the age range considered for the study) [16].

#### 2.2.2. Exclusion Criteria

- Pregnancy/desire for pregnancy;
- Uncontrolled hypertension with pharmacological treatment or decompensated cardiological diseases;
- Other chronic lung diseases;
- Inability to walk for 30 consecutive minutes without assistance;
- Cognitive impairment;
- Institutionalized patients;
- Patients with severe and poorly controlled asthma: forced expiratory volume in one second (FEV1) < 1200 mL and/or ACQ-5 > 1.5;
- Do not sign informed consent;
- Do not have a smartphone or pedometer.

### 2.3. Recruitment and Enrolment

Consecutive patients from the allergy clinic who fulfilled all specified inclusion criteria and did not meet any of the exclusion criteria were recruited. Participants who walked < 7000 steps/day were enrolled in the study. This is referred to as Visit 0 and will be detailed further in a subsequent section.

### 2.4. Randomization and Allocation

All patients were randomly assigned to 2 groups: one intervention group (IG) and one control group (CG). Recruitment was conducted by all allergists. As this was an open-label study, all investigators, in addition to the patients themselves, were aware of each subject’s group assignment. Simple randomization was carried out only by the principal investigator using sequentially numbered opaque sealed envelopes.

### 2.5. Description of the Intervention

This is a study consisting of 3 visits and a workshop with 3 sessions. Both workshop and visits took place at the Rey Juan Carlos University Hospital. In all workshop sessions, a PowerPoint presentation was used as the audiovisual support. The workshop sessions were in-person, but participants had the option to attend the final session online via the Zoom application.

The intervention structure consisted of an initial screening of patients who met the established inclusion and exclusion criteria (Visit 0), followed by confirmation that they met the required physical activity limit (<7000 daily steps). Patients who successfully completed these two steps (*n* = 52) were then randomized into two groups: the IG (*n* = 26) and the CG (*n* = 26). From this point, the intervention phase itself began, involving two visits for all patients and three workshop sessions for the IG. A more detailed description of the intervention is provided below.

- Visit 0: This visit served as the patient recruitment stage. The measurements obtained at this time represent the baseline data for all enrolled patients, recorded prior to the commencement of the intervention. For patients who were eligible a priori, spirometry with bronchodilation testing was performed, and they were administered the IPAQ-SF, ACQ-5, and mini-AQLQ. Additionally, their sociodemographic data and asthma staging were collected. Patients who met all inclusion and exclusion criteria were invited to download the previously specified pedometer app. Those who had a pedometer could use their own device. All participants were asked to complete a one-week trial during which their usual daily step average was measured. Those who walked < 7000 steps/day were enrolled in the study. All patients were randomly assigned to IG or CG. The step count from the trial week represented the baseline values for the study.
- Session 1 (start of the intervention): Only patients from the intervention group (IG) participated in the educational sessions of the workshop. The first workshop session lasted 40 min and aimed to provide an understanding of asthma as a disease and the foundations underpinning the study. To achieve this, a brief overview was provided on the pathophysiology of asthma and the causes of dyspnea and bronchospasm, especially those linked to physical exercise. Participants were informed about the WHO recommendations on physical activity and, consequently, the rationale behind the study’s proposed goal of exceeding 7000 steps daily. Finally, a 10 min group walk was conducted on the hospital grounds to increase familiarity with the application’s features and to resolve any eventual questions. From that day on, patients in both groups began daily step counting. Weekly, the principal investigator requested each patient to report their average steps, which could be sent from the participant’s app or pedometer to a mobile device enabled for the study. That report was not a self-reported measure, but the log/data generated directly by the application or the pedometer. These measurements are considered as outcomes of the intervention.
- Session 2 (IG): One week after session 1. This second session lasted 30 min. Information was provided regarding the importance of warm-up and cool-down in relation to physical exercise, the definition of exercise intensity ranges, and the definitions of aerobic and anaerobic physical exercise. Fears were collected, and questions regarding difficulties related to the goal set in the previous session were addressed.
- Session 3 (IG): One month after session 1. The third workshop lasted 20 min and was the most participatory. Participants were encouraged to discuss their experience with the program, including their subjective perception of well-being and health level after completing the intervention. Finally, a brief presentation offered advice for maintaining long-term goals.
- Visit 1 (IG and CG): One month after the start of the intervention. Spirometry with a bronchodilator test was performed, and ACQ-5 and mini-AQLQ were collected. The IG received feedback on the physical activity they were engaging in.
- Visit 2 (IG and CG): Two months after visit 1. This visit was the same as visit 1, and the study is considered completed.

The CG was asked to continue with their usual routine, without receiving instructions on changes in physical activity. The CG received the same visits as the IG but did not participate in any of the workshop sessions.

### 2.6. Outcomes

The primary outcome (asthma control) was measured using the ACQ-5. Secondary outcomes were measured using a variety of tools. The Mini-Asthma Quality of Life Questionnaire (mini-AQLQ) was used to assess asthma-related quality of life [17,18]. FEV-1 was used as the primary measure of lung capacity, in line with the majority of studies of this type. It was considered a complete, reliable and easily analyzable marker of pulmonary function: it is a well-known parameter among physicians treating respiratory pathology, including primary care physicians. Its straightforward and very clear interpretation makes it a useful and routine asthma control tool, and it is the most commonly used parameter for assessing short-, medium-, and long-term changes in respiratory function. For these reasons no other spirometric parameters were collected. Other collected variables included age, sex, body mass index (BMI), and severity of asthma (intermittent, mild and moderate persistent) [14] at the time of recruitment; the number of monthly exacerbations [14] and weekly use of rescue inhaled medication (Short-Acting Beta Agonists [SABA], combinations of corticosteroids-Long-Acting Beta Agonists [LABA] in extra doses). Exacerbations were measured using clinical records from the Primary Care Physician or Emergency Department when available, and via patient self-report when the patient did not attend a healthcare facility. The average weekly steps were measured using the mobile application Pedometer (version 2.2.159A) or with the patient’s personal pedometer. As a fundamental requirement, each subject was asked not to change the measuring device. The measurements of the variables taken at the time of recruitment are considered as baseline values for the study. The average number of steps was the only parameter used to measure physical activity. Patients were not encouraged to engage in other physical activities. The definition of unstructured physical activity means that patients were free to organize their walking according to their daily routine. No specific days, hours, or times were imposed for the performance of the physical activity.

### 2.7. Sample Size Calculation

The sample size was calculated considering a clinically relevant decrease in the ACQ-5 score of 0.74 in the intervention group and 0.34 in the control group [19], assuming an alpha risk of 0.05, a power of 80%, and a possible 10% loss; the final result is 26 patients in the IG and 26 patients in the CG. A difference of 0.5 points in the ACQ-5 (primary variable) is considered clinically relevant [11].

### 2.8. Statistical Analysis

The statistical analysis was conducted using the SPSS statistical software (IBM Statistics 20.0.1). The distribution of the variables was checked using the Kolmogorov–Smirnov test. The established confidence interval is 95%, with a significance level of *p* < 0.05. Statistical inference for repeated measures within each group was calculated using the paired *t*-test for variables with a normal distribution (age, BMI, FEV1, mini-AQLQ and steps); the Wilcoxon test was used for those variables that do not follow a normal distribution (ACQ-5, rescues, exacerbations).

For the primary analysis of the intervention effect, the change in the main outcome variables (ACQ-5, mini-AQLQ, FEV1, steps, exacerbations, and rescue medications) across the study was assessed using distinct approaches:

1. Direct comparison of change over time: A two-way Repeated Measures Analysis of Variance (ANOVA) was performed on all primary outcome variables to directly compare the change in these variables over the study period between the two study groups (Intervention vs. Control).
2. Comparison of difference scores adjusted for baseline: A “difference” variable (Final value minus Initial value) was first calculated for each outcome variable to assess the progression within each group. Subsequently, these new “difference” variables were compared between the two groups. Given the significant baseline difference observed in some key outcomes (e.g., ACQ-5), an Analysis of Covariance (ANCOVA) was primarily used for this comparison. The ANCOVA models adjusted the mean difference scores using the corresponding baseline value of the outcome variable as a covariate, thereby accounting for initial group heterogeneity. For comparison between groups without adjustment, Student’s *t*-test for independent samples was used for normally distributed differences, and the Mann–Whitney U test (Wilcoxon-Mann–Whitney) was used for the remaining variables.

Effect size measures have been included: Cohen’s *d* was reported for parametric tests, the nonparametric *r* for nonparametric tests, Cramer`s V for categorical variables and partial eta squared (η^2^_p_) for ANOVA and ANCOVA models.

Initial and final results were stratified by sex, BMI, and age. Finally, multiple linear regression models were performed, using the differences in ACQ-5 scores, mini-AQLQ, FEV1, number of exacerbations, and use of rescue medications between the start and end of the study as dependent variables. Demographic, clinical, and physical activity variables considered in the study were included as independent variables. The same multiple linear regression models were used for the values recorded at the beginning of the study in both groups, in order to assess whether the influence of the predictor variables had changed before and after the intervention.

### 2.9. Ethical Considerations

It has been conducted following the guidelines of Good Clinical Practice and the principles of the Declaration of Helsinki. It was approved on 25 April 2023 by the Clinical Research Ethics Committee of the Jiménez Díaz Foundation (EC075-23_HRJC), and all patients received and signed the corresponding written informed consent.

## 3. Results

### 3.1. Participant Flow

**Figure 1:**
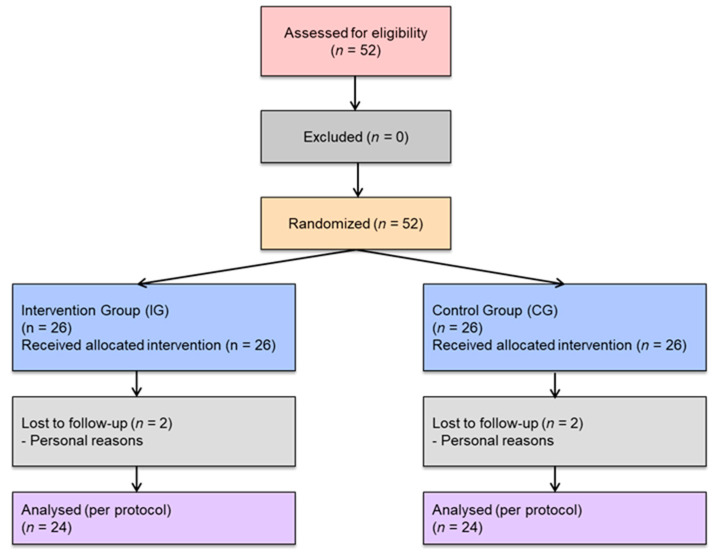
CONSORT flowchart of participants.

A total of 26 patients were recruited for each group (*n* = 52), with a dropout rate of 7.69% (4 subjects, 2 from each group, all for personal reasons), resulting in a final sample size of *n* = 48 (Figure 1). The clinical trial concluded after the study protocol was fully executed. No adverse events were observed during the study.

### 3.2. Baseline Characteristics

The characteristics of the sample are summarized in Table 1. The mean age was 36.2 years (±8.8), with a range of 19 to 50 years. Both groups were homogeneous, except regarding the distribution of sex and ACQ-5 scores between the IG and CG: men were significantly more represented in the CG, with 11 men (42.3%) compared to 3 (11.5%) in the IG (*p* = 0.012), showing a moderate effect (V = 0.3); the ACQ-5 score was significantly higher (indicating worse asthma control) in the IG, with a median of 1.2 (IQR 0.6) compared to 0.6 (IQR 1) in the CG (*p* = 0.007), also demonstrating a moderate effect (*r* = 0.3). A significantly higher average number of initial steps was also recorded in the CG, at 5043.3 (±1566) compared to the IG, which had an average of 3951 (±1471.3) steps (*p* = 0.012), with a moderate to large effect (*d* = 0.7).

When stratifying the sample by sex, age, and BMI (Table 2), a significantly higher initial quality of life was observed in patients with normal weight (*p* = 0.03), and a significantly higher initial FEV1 in men compared to women (*p* < 0.001). No other significant differences in the initial variables were found in the stratification.

### 3.3. Progression of Each Group Throughout the Study

The analysis of the evolution of the variables throughout the study for both groups is described in Table 3. A significant reduction in ACQ-5 scores (*p* = 0.03) was observed in the IG between the beginning and the end of the intervention, indicating improved asthma control. In addition to being statistically significant, this improvement is also clinically relevant (>0.5 points). The effect size was moderate-to-big (*r* = 0.4). Similarly, a significant improvement was found in mini-AQLQ (*p* = 0.01), and in the average number of steps both one-month post-intervention (*p* = 0.002) and at three months (*p* = 0.001) for the IG. The effect size was moderate for mini-AQLQ change (*d* = 0.5) and large for step count (*d* = 0.7 and *d* = 1 at 1 and 3 months, respectively). For the CG, a significant decline in FEV1 was observed throughout the study (*p* = 0.006), with a moderate effect (*d* = 0.4). In the IG, there was a decrease in the number of exacerbations and rescues, clinically relevant but not statistically significant (*p* = 0.06).

### 3.4. Comparison of the Evolution of the Variables Between IG and CG

#### 3.4.1. Change Comparison Adjusted for Baseline (ANCOVA)

To account for the baseline heterogeneity observed in ACQ-5 and step count, Analysis of Covariance (ANCOVA) was performed on the ‘difference’ variables (Final minus Initial score), using the baseline value of the respective outcome as a covariate (Table 4). The ANCOVA demonstrated that the IG achieved a significantly greater improvement than the CG only in measures of:

- Airway Function: (FEV1): The change in FEV1 was significantly more favorable in the IG (*p* = 0.04), indicating that the IG improved lung function compared to the decline observed in the CG.
- Physical Activity (Steps): The IG showed a significantly greater increase in daily steps compared to the CG (*p* = 0.01).

#### 3.4.2. Evolution over Time (Repeated Measures ANOVA)

A two-way Repeated Measures ANOVA was performed to examine the unadjusted group × time interaction for all variables (Table 5). This analysis showed a significant difference in the evolution between groups for FEV1 (*p* = 0.027), rescue medications (*p* = 0.03), and daily steps (*p* = 0.001). The change in ACQ-5 scores showed a trend toward significance in the interaction, though it did not meet the alpha < 0.05 threshold (*p* = 0.06).

#### 3.4.3. Descriptive Comparison

Consistent with the adjusted and unadjusted results, the IG achieved a significantly better asthma control (lower ACQ-5 score) when comparing its final versus initial score (*p* = 0.03). Regarding FEV1, the IG slightly improved its FEV1, while the CG experienced a statistically significant deterioration (*p* = 0.02). Similarly, throughout the study, the IG walked significantly more than the CG (*p* = 0.001). Finally, the weekly use of rescue medications throughout the study was significantly lower in the IG than in the CG (*p* = 0.035) (Table 6).

### 3.5. Subgroup and Regression Analysis

As with the initial values of the different variables, a stratification by sex, BMI, and age was also performed for their evolution throughout the study, finding no statistically significant differences (Table 7).

To analyze the influence of the sociodemographic and clinical characteristics of the patients on the effectiveness of the intervention, multiple linear regression models were developed for each of the dependent variables (Table 8). None of the independent variables showed a significant influence on the different dependent variables (*p* > 0.05).

However, when evaluating the influence of the same independent variables on the values of the dependent variables before the intervention, it is observed that BMI, the number of steps, and asthma severity do influence quality of life (mini-AQLQ) at the start of the study (*p* < 0.05), and initial FEV1 is significantly influenced by sex (*p* < 0.001) (Table 9). The aim of this regression was to assess whether any significant independent variable at the beginning of the study had lost its influence throughout the trial.

## 4. Discussion

### 4.1. Rationale and Study Design

This study evaluates the effectiveness of a group health education program promoting physical exercise in sedentary adults with asthma. Most research on exercise and asthma is heterogeneous, with conflicting results and limited evidence, which is seldom reflected in international guidelines [14]. Existing programs are often costly and prone to high dropout rates, limiting their applicability in routine clinical practice. To address these limitations, our study introduced an educational intervention encouraging unstructured walking to help patients integrate physical activity into their daily routines.

The target of 7000 daily steps was selected to identify sedentary patients, aligning with WHO recommendations for a healthy lifestyle. This specific threshold was considered appropriate for our target population: asthmatic patients who, despite being sedentary, are capable of leading a normal life without major physical or social impediments. In accordance with these guidelines, subjects were set the goal of achieving at least 7000 daily steps. While some prior studies have explored similar approaches, the evidence remains limited and partial.

### 4.2. Baseline Characteristics and Potential Confounding Factors

The study recruited more women than men, which is consistent with other studies [4,18,19,20,21] and the higher asthma prevalence among adult women [22]. However, the CG had more men, likely due to simple randomization. Initial data showed the IG had poorer asthma control (ACQ-5: 1.1 vs. 0.7 in CG) and lower activity levels (average steps), suggesting the IG was less active and had worse control at baseline.

These differences may reflect the higher proportion of women in the IG, as literature suggests lower physical activity in women can impact asthma control [3,21]. However, stratification by sex revealed no significant differences in steps or asthma control between men and women. Further analysis assessed whether the better control in the CG was related to its higher average step count. Nevertheless, multiple linear regression of the initial data did not find any influence of average steps on the ACQ-5, suggesting this baseline relationship may be attributable to chance.

Generally, the higher number of women in the IG does not appear to influence the dependent variables, as seen in both sex stratification and multiple linear regression. The only variable influenced by the higher number of women is FEV1, which is predictable since sex is a biological variable that defines an individual’s theoretical FEV1, and the female sex has a lower expected FEV1 than the male sex, independently of height and weight.

Stratification by age showed no significant findings, consistent with current literature [17,19,20]. However, patients with normal weight had a better quality of life (mini-AQLQ scores) compared to those with overweight or obesity, which aligns with previous studies [23]. Regression analysis confirmed that a higher BMI is associated with a lower quality of life, at least before the intervention. Additionally, asthma severity influenced quality of life: intermittent asthma was linked to better scores compared to mild or moderate persistent asthma. This highlights that transitioning from intermittent to persistent asthma worsens quality of life, but once persistent asthma is established, further differences in severity do not significantly impact this outcome. Few studies have explored intermittent asthma, making this an important observation.

### 4.3. Intervention Outcomes and Statistical Analysis

Given the baseline differences between the intervention and control groups (specifically the significantly worse asthma control observed in the IC), we realized different types of analyses in order to minimize the impact of sample heterogeneity.

The ANCOVA model, which evaluates change in variables over time adjusted for baseline values, demonstrated that pulmonary function (FEV1) significantly improves in the IG compared to the CG, showing a moderate-to-large effect (***η^2^_p_*** = 0.9). The same outcome was observed for the average number of steps, which significantly increased with a large effect in the IG.

When examining the unadjusted ANOVA model, which compares the direct evolution of variables over time, we observed the same positive results. This unadjusted analysis also revealed a significant reduction in rescue medication use in the IG compared to the CG (with a moderate-to-large effect). Regarding the ACQ-5, it showed a trend towards improvement with a moderate-to-large effect, but did not reach statistical significance (*p* = 0.06).

A simpler analysis comparing the differences in evolution between both groups showed that the improvement in ACQ-5, FEV1, and the average number of steps, as well as the reduction in rescue medication use, were all significant. The effect sizes ranged from small-to-moderate (ACQ-5 and rescue medication use) to moderate-to-large (average number of steps and FEV1).

The discrepancy between the unadjusted and adjusted analyses for ACQ-5 scores and rescue medication use suggests that the baseline heterogeneity-specifically the significantly worse asthma control observed in the IG at the start-likely acted as a confounding factor. This potential confounding indicates that the large unadjusted improvement in ACQ-5 in the IG might be partially attributable to the phenomenon of regression to the mean, or simply having more ‘room’ to improve.

Furthermore, the relatively small sample size may have contributed to insufficient statistical power to detect a significant difference in outcomes that showed a clear positive trend, such as ACQ-5 and exacerbations. Although the improvement in ACQ-5 did not reach statistical significance after adjustment (*p* = 0.06), the moderate effect size (*η^2^_p_* = 0.07) suggests a potential clinical relevance that warrants further investigation. Similarly, the reduction in rescue medication use did not reach statistical significance after adjustment (*p* = 0.065), but the moderate effect size (*η^2^_p_* = 0.07) indicates a change that may still be clinically meaningful. In addition, the three-month duration of the intervention may have been too short to induce or fully capture clinically relevant changes in variables that typically require more time for adaptation, such as the total number of exacerbations.

### 4.4. Discussion of Key Findings

What the study appears to evidence is that this type of intervention may influence the improvement of pulmonary function, besides being effective in promoting physical activity.

#### 4.4.1. Pulmonary Function (FEV1)

A critical consideration is the FEV1 decrease observed in the control group. Many patients in the study have allergic asthma. It is plausible that seasonal changes, with the resulting pollination peaks, may have influenced the negative change in pulmonary function in this group. It is possible that the control group contained more patients with a seasonal component to their asthma, and that this influenced the obtained results.

Few studies on educational interventions for asthma have assessed lung capacity, and results have been mixed. In the few studies focused on educational changes in physical activity for asthma (all involving unsupervised or semi-supervised exercise), either no spirometric parameters were considered [19,20,22], or the variation was not significant [24]. Even in studies with supervised and structured physical activity, a significant improvement in FEV1 has only been demonstrated in some cases [1,4]. To our knowledge, this is among the first trials to demonstrate a significant improvement in FEV1 from an unsupervised physical exercise program.

#### 4.4.2. Physical Activity and Adherence

The increase in physical activity in both the short and medium term, as a result of the intervention, is consistent with other studies that promote an active lifestyle [20,22], involving both unsupervised and semi-supervised exercise [19,24], suggesting that costly, multidisciplinary interventions are unnecessary to achieve lifestyle changes. Furthermore, walking, as measured by average steps, was more strongly associated with improved asthma control (ACQ-5) than exercise intensity, aligning with prior research [20,21]. We also consider it an interesting finding that this type of intervention had a very low dropout rate compared to studies that involve complex physical exercise programs.

#### 4.4.3. Asthma Control and Quality of Life (ACQ-5 and Mini-AQLQ)

Previous studies have shown an improvement in either quality of life or asthma control [7,24], but not in both, as evidenced by a recent systematic review [6] and a meta-analysis [4]. By performing a deeper analysis of the intervention group, we observe that both asthma control and quality of life significantly improve in this group, although the improvement in quality of life is not clinically relevant (<0.5 points). Asthma control and quality of life are complementary but independent metrics, emphasizing the need to evaluate both in asthma management. While this significance is lost when compared to the control group-despite maintaining a favorable trend-we consider this an interesting finding that warrants consideration, suggesting the need for further studies with a larger sample size and longer follow-up.

Regarding the reduction in rescue medication use, it marks a trend towards improvement, even though robust statistical significance was not reached in this trial. While prior research shows mixed results, some studies have noted reduced exacerbations [3,19] or rescue medication use [6] with increased physical activity. However, this data is not always reported or does not consistently prove to be significant.

#### 4.4.4. Other Influencing Factors and Unique Aspects

The post-intervention multivariate analysis found no significant influence of clinical or demographic variables on the main outcomes. Factors such as asthma severity, BMI, and initial activity levels-which did influence baseline quality of life-lost their significance after the intervention. This suggests that factors other than traditional clinical or demographic variables may be driving the improvements in asthma control, quality of life, and medication use. Beyond the direct physiological benefits of physical exercise on respiratory and cardiopulmonary function [25,26], the increased physical activity promoted by the intervention may facilitate broader, healthier lifestyle changes, such as smoking cessation or weight loss, which collectively enhance asthma management [20]. Furthermore, the decision to strive for a more active lifestyle and the resulting short-term increase in physical activity may enhance patients’ self-efficacy. This improved self-efficacy could lead to better management of a chronic illness like asthma, consequently impacting the number of exacerbations and rescue medication use [20].

This clinical trial stands out for including patients with intermittent asthma and those with controlled or partially controlled asthma, contrasting with most studies that focus on persistent asthma, and/or a mix of controlled, partially controlled and uncontrolled asthma [19,20,21,22]. Previous research [19] has suggested that interventions may be less effective for patients with milder asthma, but this study contradicts that assumption. The findings demonstrate that even patients with favorable clinical characteristics benefit from educational programs promoting unstructured physical activity.

#### 4.4.5. Limitations

However, the study has limitations. We acknowledge that the baseline differences, especially in asthma control, between the intervention and control groups could introduce a potential for confounding. While we cannot entirely exclude the effect of regression to the mean, our decision to analyze the within-group changes, the difference-in-differences between the groups, and adjust the results for the baseline values aims to provide a more robust comparison of the intervention’s effect. Nevertheless, the relatively small sample size may have reduced the statistical power to detect modest between-group effects, even after adjusting for baseline heterogeneity. The possibility of a Hawthorne effect should also be considered, due to the impossibility of blinding the trial. The control group was informed of their average steps, which could have led to a change in their behavior. Nevertheless, no significant rise in the average daily step count was observed in this group during the study. Furthermore, two key measurement limitations should be noted. Firstly, the use of different pedometers may result in variations in measurement accuracy, although all patients maintained the same measurement system throughout the entire trial. Secondly, reports of exacerbations and rescue medication use, when the patient did not attend the Emergency Department or visit their Primary Care physician, were based on self-reports. The 12-week duration, while average for such studies, is insufficient to evaluate long-term effects. Further research is needed to determine whether these interventions can sustain lifestyle changes and improve outcomes over time. Additionally, the results are not generalizable to patients with severe or uncontrolled asthma.

## 5. Conclusions

In conclusion, a simple, low-cost health education intervention focused on increasing daily steps is a feasible and effective strategy for improving FEV1 and physical activity in sedentary patients with asthma, including those with milder disease. Furthermore, it also appears promising in improving asthma control and quality of life, and decreasing the amount of rescue medication, but further large-scale, long-term trials are warranted to confirm these sustained effects. Finally, the minimal resources required for such programs make them feasible for implementation in primary care settings.

## Figures and Tables

**Table 1:** Description of the sample and comparison of the characteristics and initial variables of both groups.

	Total (*n* = 52)	Intervention Group (*n* = 26)	Control Group (*n* = 26)	*p* (95% CI)	(Cohen’s *d*) [*r*]
Age (years): mean (SD)	36.2 (8.8)	35.7 (8,8)	36.7 (8.9)	0.6	(0.1)
Min-max	19–50	19–50	19–50		
Sex				**0** **.** **012**	0.3 *
Male	14 (26.9%)	3 (11.5%)	11 (42.3%)		
Female	38 (73.1%)	23 (88.5%)	15 (57.7)		
BMI: mean (SD)	25.1 (4.3)	25.5 (5.1)	24.8 (3.3)	0.5	(0.1)
Min-max	18–35	18–35	18–32		
Asthma				0.52	0.1 *
Intermittent	18 (34.5%)	10 (38.5%)	8 (30.8%)		
Mild persistent	22 (42.3%)	9 (34.6%)	13 (50%)		
Moderate persistent	12 (23.1%)	7 (26.9%)	5 (19.2%)		
FEV1 (L): mean (SD)	3.27 (0.6)	3.1 (0.6)	3.4 (0.6)	0.06	(0.5)
ACQ-5: median (IQR)	0.9 (1)	1.2 (0.6)	0.6 (1)	**0.007**	**[0.3]**
AQLQ: mean (SD)	5.4 (0.9)	5.4 (0.7)	5.4 (1.2)	0.9	(0.01)
Exacerbations:				0.19	[0.1]
mean (SD)	0.1 (0.5)	0.3 (0.7)	0.08 (0.28)		
Rescues: mean (SD)	0.2 (0.6)	0.4 (0.8)	0.2 (0.6)	0.059	[0.2]
Mean daily steps (SD)	4478 (1644)	3951.2 (1471.3)	5043.3 (1566)	**0.012**	**(0.7)**

* Cramer’s V.

**Table 2:** Stratification of the initial variables by age, BMI, and sex.

	19–35 Years	36–50 Years	*p*
Initial ACQ-5: median (IQR)	1.2 (0.8)	0.8 (0.8)	>0.05
Mean (SD)	1.1 (0.9)	0.7 (0.5)	
Initial Mini-AQLQ: mean (SD)	5.3 (1)	5.5 (0.9)	>0.05
Initial FEV1 (L): mean (SD)	3.2 (0.4)	3.2 (0.6)	>0.05
Initial daily steps: mean (SD)	4494 (1442)	4488 (1739)	>0.05
Initial rescues: mean (SD)	0.4 (0.6)	0.3 (0.7)	>0.05
Initial exacerbations: mean (SD)	0.1 (0.5)	0.2 (0.5)	>0.05
	Normal Weight *	Overweight/Obesity *	***p***
Initial ACQ-5: median (RIQ)	1 (0.8)	0.9 (1)	>0.05
Initial mini-AQLQ: mean (SD)	5.7 (0.9)	5.1 (0.9)	**0** **.** **03**
Initial FEV1 (L): mean (SD)	3.2 (0.5)	3.1 (0.7)	>0.05
Initial daily steps: mean (SD)	4602 (1487)	4358 (1775)	>0.05
Initial rescues: mean (SD)	0.3 (0.7)	0.3 (0.7)	>0.05
Initial exacerbations: mean (SD)	0.07 (0.2)	0.3 (0.7)	>0.05
	**Male**	**Female**	***p***
Initial ACQ-5: median (IQR)	0.6 (0.8)	1 (0.9)	>0.05
Initial Mini-AQLQ: mean (SD)	5.7 (0.9)	5.3 (0.9)	>0.05
Initial FEV1 (L): mean (SD)	3.8 (0.4)	3 (0.4)	**<0** **.** **001**
Initial daily steps: mean (SD)	4711 (1642)	4417 (1619)	>0.05
Initial rescues: mean (SD)	0.09 (0.7)	0.1 (1.7)	>0.05
Initial exacerbations: mean (SD)	0.08 (0.2)	0.4 (0.8)	>0.05

* Normal weight: BMI < 25; overweight/obesity: BMI ≥ 25.

**Table 3:** Evolution of the clinical variables and the number of steps in the intervention group and control group.

Intervention Group	Initial (*n* = 24)	Final (*n* = 24)	*p* (95% CI)	(Cohen’s *d*) [*r*]
FEV1 (L): mean (SD)	3.1 (0.6)	3.1 (0.6)	0.55	(0.1)
Mini-AQLQ: mean (SD)	5.4 (0.7)	5.8 (0.8)	**0** **.** **017**	**(0.5)**
ACQ-5: median (IQR)	1.2 (0.6)	0.6 (1)	**0** **.** **035**	**[0.4]**
Exacerbations: mean (SD)	0.3 (0.8)	0.04 (0.2)	0.06	(0.3)
Rescues: mean (SD)	0.4 (0.6)	0.1 (0.3)	0.058	(0.1)
Daily steps at 1 month: mean (SD)	3857 (1553)	5167 (1835)	**0** **.** **002**	**(0.7)**
Daily steps at 3 months: mean (SD)	3857 (1553)	5623 (1788)	**<0** **.** **001**	**(1)**
**Control group**				
FEV1 (L): mean (SD)	3.3 (0.5)	3.2 (0.6)	**0** **.** **006**	**(0.6)**
Mini-AQLQ: mean (SD)	5.5 (1)	5.7 (0.9)	0.39	(0.1)
ACQ-5: median (IQR)	0.6 (1)	0.6 (1)	0.3	[0.2]
Exacerbations: mean (SD)	0.07 (0.2)	0.09 (0.2)	0.31	[0.2]
Rescues: mean (SD)	0.3 (0.6)	0.9 (2)	0.14	[0.3]
Daily steps at 1 month: mean (SD)	5124 (1436)	5528 (1837)	0.17	(0.2)
Daily steps at 3 months: mean (SD)	5124 (1436)	5527 (1800)	0.15	(0.2)

**Table 4:** Analysis of Covariance (ANCOVA) Results for Change Scores, Adjusted for Baseline Values.

Source of Variation	d*f*	MS	F	*p*	*η^2^_p_*
Baseline **ACQ-5** (covariate)	1	4.7	7.3	0.01	0.1
Group	1	1.1	1.7	0.1	0.038
Error	44	0.6			
Baseline **FEV1** (covariate)	1	0.01	0.28	0.5	0.007
Group	1	0.2	4.4	**0.04**	**0.09**
Error	42	0.04			
Baseline **rescues** (covariate)	1	7.7	3.5	0.06	0.07
Group	1	7.9	3.7	0.06	0.07
Error	44	2.1			
Baseline **Mini-AQLQ** (covariate)	1	16.7	22.6	<0.001	0.3
Group	1	0.3	0.4	0.5	0.01
Error	44	0.7			
Baseline **Exacerbations** (covariate)	1	11.1	174.5	<0.001	0.8
Group	1	0.04	0.6	0.4	0.01
Error	43	0.06			
Baseline **steps** (covariate)	1	3,922,351.9	2.1	0.1	0.04
Group	1	12,587,980.8	6.9	**0.01**	**0.1**
Error	45	1,810,362.1			

d*f*: degrees of freedom; MS: mean square; *η^2^_p_* partial eta square.

**Table 5:** Analysis of Variance (ANOVA) for Main Variables Across the Study.

Source of Variation	SS	d*f*	MS	F	*p*	*η^2^_p_*
**ACQ-5**						
Time	0.3	1	0.3	0.9	0.3	0.02
Time x group	1.3	1	1.3	3.5	0.06	0.07
Error (group)	16.6	45	0.3			
**FEV1**						
Time	0.04	1	0.04	1.7	0.19	0.03
Time x group	0.12	1	0.12	5.2	**0.027**	**0.10**
Error (group)	1	43	0.02			
**Rescues**						
Time	0.,7	1	0.7	0.6	0.4	0.01
Time x group	5.2	1	5.2	4.6	**0.03**	**0.09**
Error (group)	51	45	1.1			
**Mini-AQLQ**						
Time	2.5	1	2.5	4.6	0.03	0.09
Time x group	0.2	1	0.2	0.4	0.4	0.01
Error (group)	24.6	45	0.5			
**Exacerbations**						
Time	0.08	1	0.08	0.6	0.4	0.01
Time x group	0.2	1	0.2	1.8	0.1	0.04
Error (group)	6.1	45	0.1			
**Steps**						
Time	28,218,159.3	1	28,218,159.3	300.4	<0.001	0.39
Time x group	11,152,073.4	1	11,152,073.4	12	**0.001**	**0.2**
Error (group)	42,694,323.7	46	928,137.4			

SS: sum of squares; d*f*: degrees of freedom; MS: mean square; *η^2^_p_:* partial eta square.

**Table 6:** Comparison of the evolution of the variables during the study between the intervention and control groups.

	Difference in the IG *	Difference in the CG *	*p* (95% CI)	(Cohen’s *d*) [*r*]
ACQ-5 **: mean (SD)	−0.3 (0.8)	0.11 (0.8)	**0** **.** **03**	**[0.3]**
Mini-AQLQ: mean (SD)	−0.4 (0.8)	−0.2 (1.2)	0.49	0.2
FEV1 (L): mean (SD)	0.03 (0.2)	−0.1 (0.18)	**0** **.** **02**	**(0.6)**
Daily steps: mean (SD)	1765 (1378)	402 (1346)	**0** **.** **001**	**(1)**
Exacerbations: mean (SD)	−0.29 (0.7)	−0.04 (0.2)	0.06	[0.1]
Rescues: mean (SD)	−0.2 (0.7)	0.6 (2)	**0** **.** **035**	**[0.3]**

* Final measurement—Initial measurement; ** Mean and SD are provided since the median is 0.

**Table 7:** Stratification by age, BMI, and sex between the beginning and the end of the study.

	19–35 Years	36–50 Years	*p*
ACQ difference: median (IQR)	0 (1.2)	0 (0.9)	>0.05
Mean (SD)	−0.2 (0.8)	−0.01 (0.8)	
Mini-AQLQ difference: mean (SD)	0.4 (0.8)	0.2 (1.1)	>0.05
FEV1 difference: mean (SD)	0.02 (0.2)	−0.08 (0.1)	>0.05
Daily steps difference: mean (SD)	1167 (1431)	1030 (1587)	>0.05
Rescues difference: mean (SD)	0.1 (1.8)	0.1 (1.3)	>0.05
Exacerbations difference: mean (SD)	−0.1 (0.5)	−0.1 (0.6)	>0.05
	**Normal weight**	**Overweight/obesity**	***p***
ACQ-5 difference: median (IQR)	0 (1)	0 (1)	>0.05
Mean (SD)	−0.1 (0.9)	−0.1 (0.8)	
Mini-AQLQ difference: mean (SD)	0.2 (1)	0.4 (0.9)	>0.05
FEV1 (L) difference: mean (SD)	−0.01 (0.2)	−0.07 (0.2)	>0.05
Daily steps difference: mean (SD)	722 (1389)	1511 (1573)	>0.05
Rescues difference: mean (SD)	0.4 (2)	−0.09 (0.7)	>0.05
Exacerbations difference: mean (SD)	0.04 (0.2)	−0.3 (0.7)	>0.05
	**Male**	**Female**	***p***
ACQ-5 difference: median (IQR)	0 (0.6)	0 (1.2)	>0.05
Mean (SD)	−0,09 (0.3)	−0.1 (0.9)	
Mini-AQLQ difference: mean (SD)	0.04 (1.3)	0.4 (0.9)	>0.05
FEV1 (L) difference: mean (SD)	−0.08 (0.2)	−0.03 (0.2)	>0.05
Daily steps difference: mean (SD)	695 (902)	1213 (1658)	>0.05
Rescues difference: mean (SD)	0.09 (0.7)	0.1 (1.7)	>0.05
Exacerbations difference: mean (SD)	−0.1 (0.6)	−0.5 (0.6)	>0.05

**Table 8:** Results of Multiple Linear Regression Analysis: Predictors of Dependent Variables (Baseline-End Difference).

	Independent Variables	*p*	Unstandardized B Coefficient	Standardized B Coefficient	*r*-Squared
ACQ-5 difference	Age	0.2	0.02	0.2	0.05
	BMI	0.5	−0.02	−0.1	
	Sex	0.9	−0.005	−0.003	
	Intermittent asthma	0.4	−0.2	−0.1	
	Mod persistent asthma	0.3	−0.3	−0.1	
	Steps difference	0.5	5.9 × 10^−5^	0.1	
Mini-AQLQ difference	Age	0.14	−0.03	−0,2	0.15
	BMI	0.07	0.08	0.3	
	Sex	0.4	0.2	0.1	
	Intermittent asthma	0.6	−0.15	−0.07	
	Mod persistent asthma	0.15	−0.5	−0.2	
	Steps difference	0.4	9.4 × 10^−5^	0.1	
FEV1 difference	Age	0.1	−0.008	−0.3	0.07
	BMI	0.7	0.004	0.07	
	Sex	0.8	0.02	0.04	
	Intermittent asthma	0.7	−0.02	−0.05	
	Mod persistent asthma	0.8	0.01	0.03	
	Steps difference	0.8	3.2 × 10^−6^	−0.02	
Exacerbations difference	Age	0.4	0.01	0.14	0.12
	BMI	0.3	−0.02	−0.2	
	Sex	0.5	0.1	0.09	
	Intermittent asthma	0.4	−0.1	−0.1	
	Mod persistent asthma	0.9	0.02	0.01	
	Steps difference	0.08	0	−0.2	
Rescues difference	Age	0.1	0.05	0.3	0.06
	BMI	0.1	−0.09	−0.2	
	Sex	0.7	0.2	0.05	
	Intermittent asthma	0.9	0.04	0.1	
	Mod persistent asthma	0.7	−0.19	−0.05	
	Steps difference	0.8	3.1 × 10^−5^	0.03	

**Table 9:** Multiple linear regression analysis results: predictors of the dependent variables at baseline.

	Independent Variables	*p*	Unstandardized B Coefficient	Standardized B Coefficient	*r*-Squared
Initial ACQ-5	Age	0.2	−0.02	−0.2	0.14
	BMI	0.2	0.03	0.2	
	Sex	0.2	0.3	0,19	
	Intermittent asthma	0.16	−0.3	−0.2	
	Mod persistent asthma	0.8	0.04	0.02	
	Initial steps	0.8	−9.2 × 10^−6^	−0.02	
Initial mini-AQLQ	Age	0.11	0.03	0.27	0.3
	BMI	**0** **.** **003**	−0.1	−0.5	
	Sex	0.2	−0.3	−0.1	
	Intermittent asthma	**0** **.** **02**	0.6	0.3	
	Mod persistent asthma	0.3	0.3	0.1	
	Initial steps	**0** **.** **026**	0.0	−0.2	
Initial FEV1	Age	0.4	−0.007	−0.1	0.5
	BMI	0.3	−0.017	−0.12	
	Sex	**<0** **.** **001**	−0.8	−0.6	
	Intermittent asthma	0.2	0.19	0.15	
	Mod persistent asthma	0.1	−0.	−0.19	
	Initial steps	0.9	−8.3 × 10^−7^	−0.002	
Initial exacerbations	Age	0.5	0.007	0.11	0.06
	BMI	0.6	0.013	0.1	
	Sex	0.5	0.1	0.08	
	Intermittent asthma	0.3	0.16	0.15	
	Mod persistent asthma	0.5	0.14	0.11	
	Initial steps	0.8	8.2 × 10^−6^	0.024	
Initial rescues	Age	0.5	0.007	0.11	0.1
	BMI	0.6	0.01	0.1	
	Sex	0.5	0.1	0.08	
	Intermittent asthma	0.3	0,16	0.15	
	Mod persistent asthma	0.5	0.14	0.11	
	Initial steps	0.8	8.2 × 10^−6^	0.02	

## Data Availability

The raw data supporting the conclusions of this article will be made available by the authors upon request.

## Abbreviations

The following abbreviations are used in this manuscript:

AQLQ	Asthma Quality of Life Questionnaire
BMI	Body Mass Index
CG	Control Group
FEV1	Forced Expiratory Volume in 1 s
IG	Intervention Group
IPAQ-SF	International Physical Activity Questionnaire- Short Form
LABA	Long-Acting Beta Agonists
Mini-AQLQ	Mini- Asthma Quality of Life Questionnaire
SABA	Short-Acting Beta Agonists
WHO	World Health Organization

## Appendix A

**Table A1:** CONSORT 2010 checklist.

Section/Topic	Item Nº	Checklist Item	Reported on Page Nº
Title and abstract	1a	Identification as a randomised trial in the title	1
	1b	Structured summary of trial design, methods, results, and conclusions	1
Introduction	2a	Scientific background and explanation of rationale	2
	2b	Specific objectives or hypotheses	2–3
Methods—Trial design	3a	Description of trial design (such as parallel, factorial); allocation ratio	3
	3b	Important changes to methods after trial commencement, with reasons	N/A
Participants	4a	Eligibility criteria for participants	3
	4b	Settings and locations where the data were collected	3–4
Interventions	5	The interventions for each group, with details for replication	4–5
Outcomes	6a	Completely defined pre-specified primary and secondary outcome measures	5
	6b	Any changes to trial outcomes after the trial commenced, with reasons	N/A
Sample size	7a	How sample size was determined	5
	7b	When applicable, explanation of any interim analyses and stopping guidelines	N/A
Randomisation—Sequence generation	8a	Method used to generate the random allocation sequence	4
	8b	Type of randomisation; details of any restriction (such as blocking)	4
Allocation concealment mechanism	9	Mechanism used to implement the random allocation sequence	4
Implementation	10	Who generated the allocation sequence, who enrolled participants, and who assigned participants	4
Blinding	11a	If done, who was blinded after assignment to interventions and how	N/A
	11b	If relevant, description of the similarity of interventions	N/A
Statistical methods	12a	Statistical methods used to compare groups for primary and secondary outcomes	5–6
	12b	Methods for additional analyses, such as subgroup analyses and adjusted analyses	5–6
Results—Participant flow	13a	For each group, the numbers of participants randomly assigned, treated, analysed	6
	13b	For each group, losses and exclusions after randomisation, with reasons	6
Recruitment	14a	Dates defining periods of recruitment and follow-up	3
	14b	Why the trial ended or was stopped	N/A
Baseline data	15	A table showing baseline demographic and clinical characteristics for each group	8
Numbers analysed	16	For each group, number of participants analysed and denominator for each analysis	8
Outcomes and estimation	17a	For each outcome, results for each group, and estimated effect size and precision	7–9
	17b	For binary outcomes, presentation of both absolute and relative effect sizes	N/A
Ancillary analyses	18	Results of any other analyses performed, including subgroup analyses and adjusted analyses	N/A
Harms	19	All important harms or unintended effects in each group	7–9
Discussion—Limitations	20	Trial limitations, addressing sources of potential bias, imprecision, multiplicity	15
Generalisability	21	Generalisability (external validity, applicability) of the trial findings	15–16
Interpretation	22	Interpretation consistent with results, balancing benefits and harms	16
Other information—Registration	23	Registration number and name of trial registry	3
Protocol	24	Where the full trial protocol can be accessed, if available	N/A
Funding	25	Sources of funding and role of funders	16
